# Do All Roads Lead to Rome? The Potential of Different Approaches to Diagnose *Aelurostrongylus abstrusus* Infection in Cats

**DOI:** 10.3390/pathogens10050602

**Published:** 2021-05-14

**Authors:** Katharina Raue, Jonathan Raue, Daniela Hauck, Franz Söbbeler, Simone Morelli, Donato Traversa, Manuela Schnyder, Holger Volk, Christina Strube

**Affiliations:** 1 Institute for Parasitology, Centre for Infection Medicine, University of Veterinary Medicine Hannover, 30559 Hannover, Germany; katharina.raue@tiho-hannover.de (K.R.); daniela.hauck@tiho-hannover.de (D.H.); 2 Department for Small Animal Medicine and Surgery, University of Veterinary Medicine Hannover, 30559 Hannover, Germany; jonathan.raue@tiho-hannover.de (J.R.); franz.josef.soebbeler@tiho-hannover.de (F.S.); holger.volk@tiho-hannover.de (H.V.); 3 Faculty of Veterinary Medicine, University of Teramo, 64100 Teramo, Italy; smorelli@unite.it (S.M.); dtraversa@unite.it (D.T.); 4 Institute of Parasitology, Vetsuisse Faculty, University of Zurich, 8057 Zürich, Switzerland; manuela.schnyder@uzh.ch

**Keywords:** cat lungworm, aelurostrongylosis, parasitic bronchopneumonia, diagnostic imaging, computed tomography, X-ray, serology, larvae counts

## Abstract

An infection with the cat lungworm, *Aelurostrongylus abstrusus*, can be subclinical, but it can also cause severe respiratory clinical signs. Larvae excretion, antibody levels, clinical assessment findings of the respiratory system and diagnostic imaging findings were recorded and compared for six cats with experimental aelurostrongylosis. In five cats, patency started 33–47 days post infection (pi), but two cats excreted larvae only in long intervals and low numbers. Positive ELISA results were observed in four cats with patent aelurostrongylosis, starting between five days before and 85 days after onset of patency. One seropositive cat remained copromicroscopically negative. Mild respiratory signs were observed in all cats examined. A computed tomographic (CT) examination of the lungs displayed distinct alterations, even in absence of evident clinical signs or when larvae excretion was low or negative. The thoracic radiograph evaluation correlated with the CT results, but CT was more distinctive. After anthelmintic treatment in the 25th week post infection, pulmonary imaging findings improved back to normal within 6–24 weeks. This study shows that a multifaceted approach, including diagnostic imaging, can provide a clearer diagnosis and monitoring of disease progression. Furthermore, a CT examination provides an alternative to *post mortem* examination and worm counts in anthelmintic efficacy studies.

## 1. Introduction

The cat lungworm, *Aelurostrongylus abstrusus* Railliet, 1898 (Nematoda, Metastrongyloidea), causes lower respiratory tract disease in cats worldwide [1,2,3,4,5]. After the ingestion of the infective third-stage larvae (L3) in gastropod intermediate hosts or paratenic hosts, e.g., small rodents [6,7,8,9], the L3 migrate from the small intestine to the lungs. Here they mature into small adult worms (5.0–10.4 mm in length and less than 0.1 mm in width), which reside usually in pairs or small groups in the lung parenchyma, i.e., the bronchioles, alveolar ducts and alveoli [6,9,10]. Feline aelurostrongylosis can clinically manifest as granulomatous pneumonia with clinical signs such as coughing, sneezing and nasal discharge, or, in more severe cases, tachypnea or dyspnea and apathy, stunting or even death [11,12,13,14]. However, infections may also remain without noticeable respiratory signs despite the presence of lung pathology [15,16,17]. Lungworm infections are probably underdiagnosed or misdiagnosed in both subclinically infected and cats with clinical signs, as similar respiratory signs have been associated with feline asthma or other bronchial infectious diseases, and because treatment for feline asthma often eases the clinical appearance of aelurostrongylosis [18]. Similarly, thoracic radiographs showing a multifocal nodular bronchointerstitial pulmonary pattern, increased opacity, diffuse areas of higher density, thickening of bronchial walls and sternal lymphadenopathy upon aelurostrongylosis [11,12,19,20] represent non-specific alterations with no definitive diagnosis [20,21].

The most frequent procedure for diagnosing aelurostrongylosis is the Baermann method, i.e., a non-invasive and inexpensive faecal examination. However, it allows diagnosis only during patency, and the sensitivity is impaired by intermittent and/or low excretion of larvae [13,21]. Thus, sampling over several consecutive days is recommended [22], but this procedure can, in turn, lead to lower survival rates of the first-stage larvae (L1), hampering the sensitivity of the copromicroscopical examination [23] and delaying diagnosis. In addition, the Baermann method requires up to 24 h until L1 can be found, and a trained clinical parasitologist is required to reliably identify *A. abstrusus* L1 [22]. In some cases, molecular detection by PCR using faeces [24] or mucous obtained by pharyngeal swaps [25,26] can increase diagnostic sensitivity and specificity. An advantage of PCR over Baermann detection is that PCR can work independently from the presence of L1 in faeces, as it also detects, if present in sufficient quantity, DNA molecules released from decaying cells of developing or adult lungworm stages [27]. A recently developed ELISA for the detection of antibodies against *A. abstrusus* in serum is a novel technique that can support the diagnosis of the infection [28]. This ELISA is based on recombinantly expressed major sperm protein (MSP) of the bovine lungworm *Dictyocaulus viviparus* as a diagnostic antigen [29]. When utilizing a horseradish peroxidase (HRP)-conjugated secondary antibody, sensitivity ranged from 88.2% to 100% and specificity from 85% to 92.6%, depending on the used plate type [28]. Until now, the ELISA has mainly been used in epizootiological research studies but is not commercially available for clinical diagnosis, although it is offered and performed in specialised labs [30,31,32,33]. It should be taken into account that based on the nature of the diagnostic antigen, seroconversion is dependent on the presence of adult male lungworms. Consequently, the test does not allow diagnosis of immature infections, and nor does the Baermann method. Furthermore, false-positive results can occur as antibodies can persist for at least 30 days after parasite death due to anthelmintic treatment or natural parasite elimination [28,31]. Nevertheless, the rather short persistence of anti-MSP antibodies enables a fairly timely control of treatment success.

A major drawback of almost all mentioned diagnostic tools is their inability to detect immature or prepatent infections. In this early phase of infection, lung damage has most likely occurred [34], and in untreated cats, histopathological alterations may be visible for up to 24 months after initial infection, even if postpatency is reached [34]. Interestingly, a retrospective study showed that 9% of *post mortem* examined cats, which deceased during anaesthesia, were infected with lungworms [35]. Consequently, the potentially increased anaesthetic risk justifies diagnostic procedures in apparently clinically healthy cats prior to anaesthesia. In addition to the aforementioned diagnostic tools, computed tomography (CT) examinations of the lungs could not only help in making a diagnosis but also in assessing the degree of lung damage, as it may allow quantitative analysis of the severity of lesions [19]. CT may also aid in clinical studies evaluating anthelmintic drug efficacy against *A. abstrusus*, and, in combination with other diagnostic methods, has the potential to replace post mortem examinations.

The aim of this study was to investigate the course of aelurostrongylosis in experimentally infected cats by combining clinical, faecal, serological and diagnostic imaging examinations and to correlate the different findings. Furthermore, findings obtained with the aforementioned diagnostics were also evaluated after anthelmintic treatment.

## 2. Results

### 2.1. Faecal Larvae Counts

**Figure 1:**
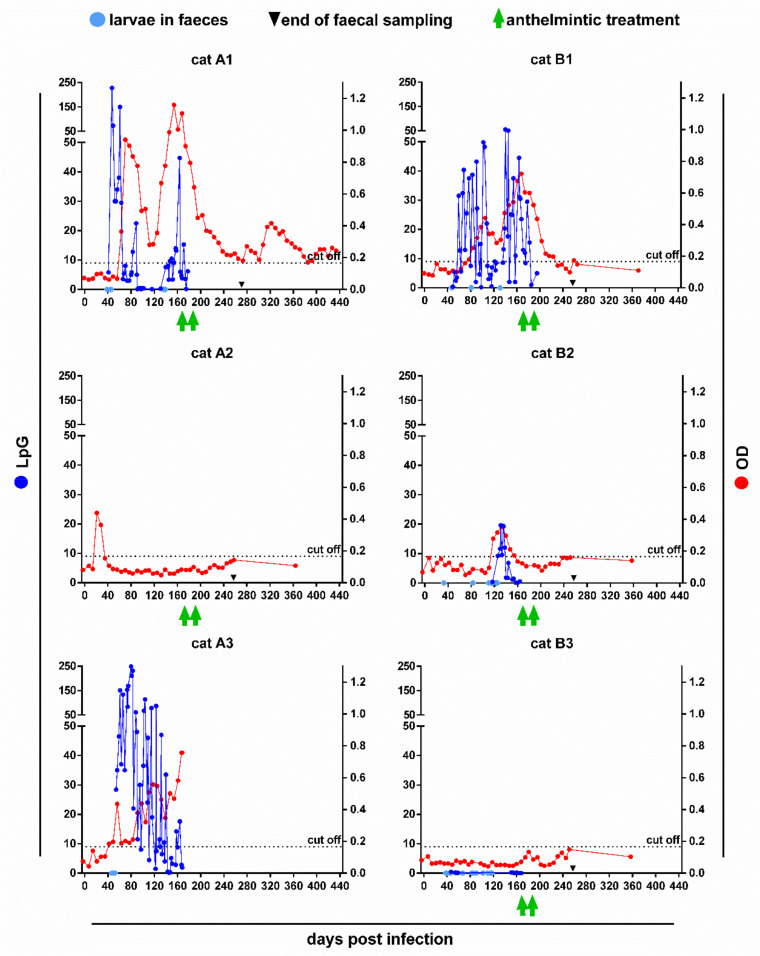
Faecal larvae excretion and anti-MSP antibody ELISA OD values in six cats experimentally infected with *A. abstrusus*. Light blue circles indicate positive faecal samples without further quantification of larvae. The dotted black line marks the ELISA cut-off value of 0.166 OD. Note that cat A3 deceased on day 168 pi.

In five of the six cats, excretion of *A. abstrusus* L1 was detected using the Baermann method, while cat A2 remained copromicroscopically negative throughout the study. The first positive results were observed between 33 and 47 days post infection (dpi; Figure 1). The maximum number of L1 per gram of faeces (LpG) ranged between 0.5 (cat B3) and 248.5 (cat A3). During patency, all cats showed periods of very low or undetectable larvae excretion. One patent cat (A3) deceased on day 168 post infection (pi) due to reasons unrelated to the lungworm infection. The last positive Baermann results of cats B2 and B3 were obtained 164 and 168 dpi, which corresponded to 7 and 3 days before the first anthelmintic treatment, respectively. In cats A1 and B1, larvae excretion was detected until 7 and 14 days after the first anthelmintic treatment.

### 2.2. Serology

Five of the six cats (A1–A3, B1–B2) exceeded the cut-off value of 0.166 optical density (OD) during the study (Figure 1). Seroconversion occurred between day 21 and 118 pi and lasted for various periods. Interestingly, cat A2 remained copromicroscopically negative throughout the study, but showed the earliest, albeit shortest, seropositivity from day 21 to 28 pi. A short period of seropositivity, corresponding to quantifiable LpG values, was also observed in the cat with the latest onset (cat B2, days 118–154 pi). Similarly, in cats A1, A3 and B1 seropositivity correlated, with some time lag, in its course and OD levels with the respective LpG levels. In cat A3, seroconversion was evident five days before the first larvae detection on day 47 pi, while cats A1, B1 and B2 turned seropositive 16–85 days after the first larvae detection. In cat B3, the OD values never exceeded the cut-off despite positive Baermann results. However, this cat excreted very low numbers of L1, reaching a maximum of 0.5 LpG only.

After anthelmintic treatment, the fast decline in larvae excretion of the high-shedders A1 and B1 was closely followed by decreasing OD values. In cat B1, they dropped below the cut-off within 60 days after the first emodepside treatment, while cat A1 remained seropositive during the subsequent sampling period of 263 days. In contrast, in the seropositive low-shedder (cat B2), detectable excretion of larvae already stopped the week before the first treatment and the OD values fell below the cut-off even a week earlier. Interestingly, in all cats, the OD values increased again from 5 to 15 weeks after the first treatment, particularly in cat A1.

### 2.3. Respiratory Parameters

**Figure 2:**
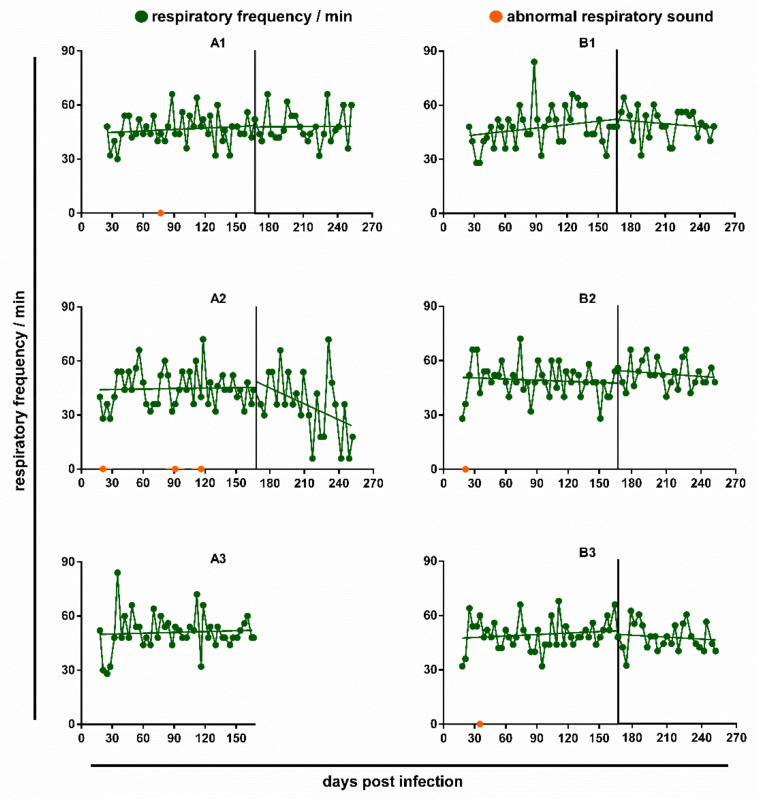
Respiratory frequency and occurrence of abnormal respiratory sounds in the study cats experimentally infected with *A. abstrusus* on day 0. Orange circles represent abnormal respiratory sounds such as rhonchus, crackle, wheeze, coughing or retching. The vertical black line marks the first anthelmintic treatment at 171/173 dpi.

In all cats, the respiratory frequency remained without significant changes during the observation period (Figure 2). However, there was a pronounced decrease in the respiratory frequency after anthelmintic treatment in cat A2, even though this individual was never found positive on the Baermann’s test.

During lung auscultation, all cats exhibited a slight to moderate increase in respiratory depth over the observation period, beginning already at day 21 pi. However, the occurrence did not correspond to the number of excreted L1. Indeed, in the low-excreting cats B2 and B3, an increase in respiratory depth was almost consistently observed until 10 or 18 weeks post infection (wpi), respectively, and even the cat without detectable larvae excretion (A2) showed this aberrance intermittently. In all cats, the alterations in the depth of respiration became already less frequent before anthelmintic treatment, but disappeared completely within four weeks after the first deworming in cats A1, A2 and B1. Additional abnormal respiratory sounds were noticed on single occasions in four cats: Cat A1 showed slight to moderate rhonchus and coughing during auscultation at day 77 pi; A2 reacted to blood sampling with a crackling respiratory sound at day 21 pi and with panting and coughing at day 91 pi, respectively, and was coughing during auscultation on day 116 pi; B2 showed retching at day 22 pi during blood sampling, and B3 had a slight wheezing sound at day 35 pi during auscultation. Notably, with the exception of cat A1, respiratory anomalies were observed prior to (B1, B2), or despite lacking (A2), larvae excretion. Detailed results of the respiratory examinations are provided in Table S1. None of the cats was impaired in general appearance or behaviour.

### 2.4. Computed Tomography

**Figure 3:**
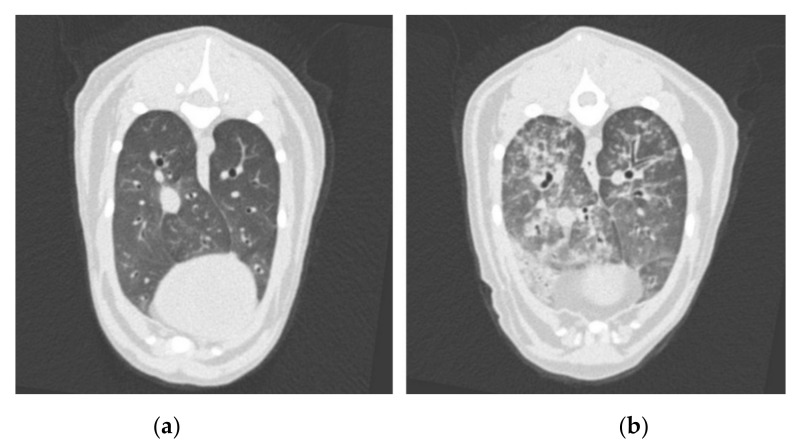
Transverse thoracic computed tomography (CT) images at the level of the caudal lung lobes showing (**a**) an unremarkable lung pattern scored as 0, and (**b**) a significantly altered lung pattern of an *A. abstrusus*-infected cat given a score of 3.

The overall severity score results of each cat on each examination day of both imaging modalities are shown in Table 1. All lung images were unremarkable (score 0 as shown in Figure 3a) on the day of infection. Lung lesions up to a severity score of 3 (Figure 3b) were found in all cats after infection in CT in the weeks 12 to 24. After anthelmintic treatment in the 25th wpi, all lungs improved on the next CT scan six weeks later. In one cat (A1), a full recovery was already reached in the 31st wpi, in three cats (A2, B1 and B2) in the 36th/37th wpi, and in one cat (B3) in the 48th wpi. The most frequently recorded changes in all CT studies after infection were a mosaic pattern and bronchial wall thickening, followed by areas of high attenuation, such as ground-glass opacity, or an increase in consolidated areas, as well as linear and reticular pattern, and isolated nodules or nodular lung patterns. Generally, a mix of patterns was found on every CT scan, but not all types of patterns were recorded in every scan. For example, one cat (B3) had only very mildly visible isolated nodules in the 25th wpi, whereas the main finding at all other time points, except the last scan in the 48th wpi, was a mixture of a linear, reticular and mosaic pattern. Despite some lung lobes being more affected than others, every lobe was affected in all CT studies, except in cat B3, where at 36 wpi, only unilaterally located lobes were altered, and at 42 wpi, only a single lobe was altered, respectively. No changes in pulmonary artery dimensions were visible on the CT, as far as can be assessed without pulmonary arterial angiography. Lymph node enlargement was detected in all cats until 0–12 weeks after anthelmintic treatment.

### 2.5. Radiography

**Figure 4:**
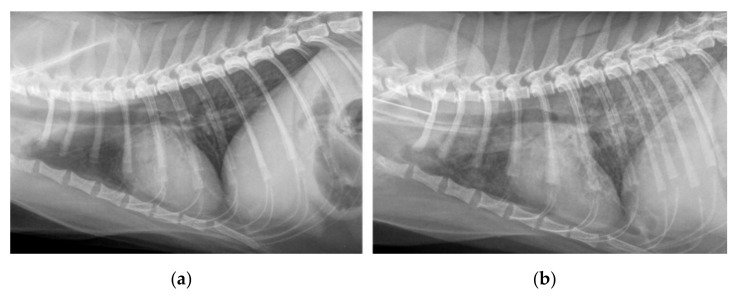
Laterolateral right-sided thoracic radiographs of a cat showing (**a**) an unremarkable lung pattern scored as 0, and (**b**) a significantly altered lung pattern of an *A. abstrusus*-infected cat given a summarized score of 7.

Similar to the CT, all lungs were scored as normal (Table 1; example image provided in Figure 4a) on the first thoracic radiograph before infection, except for cat A2 which showed a mild bronchial pattern, which was, in contrast, not visible in CT on the same day. The radiographically most affected lung, with a score of 7, was seen at 12 wpi in cat A1 (Figure 4b). The same cat remained at a score of 1 on its final radiographic examination in the 31st wpi due to a mild bronchial pattern and suspected isolated nodules, but the lung pattern was classified as unremarkable on the CT images. In all other cats, the score improved over time until it was back to normal, after the initial deterioration to an individual maximum at 12 wpi. Similar to the improvement seen on the CT, lung images improved noticeably after anthelmintic treatment in the 25th wpi. The predominant lung pattern after infection was a bronchial pattern, reaching a maximum of score 3 in three cats. In contrast, nodular lung patterns were not evident in two cats (B2 and B3) at all, despite nodules having been found on CT images. Reticular interstitial, as well as alveolar patterns of lower degrees (maximum pattern score 2), were also frequently visible. Nodular patterns with a maximum score of 1 were only occasionally identified.

### 2.6. Post Mortem Nematode Counts

**Figure 5:**
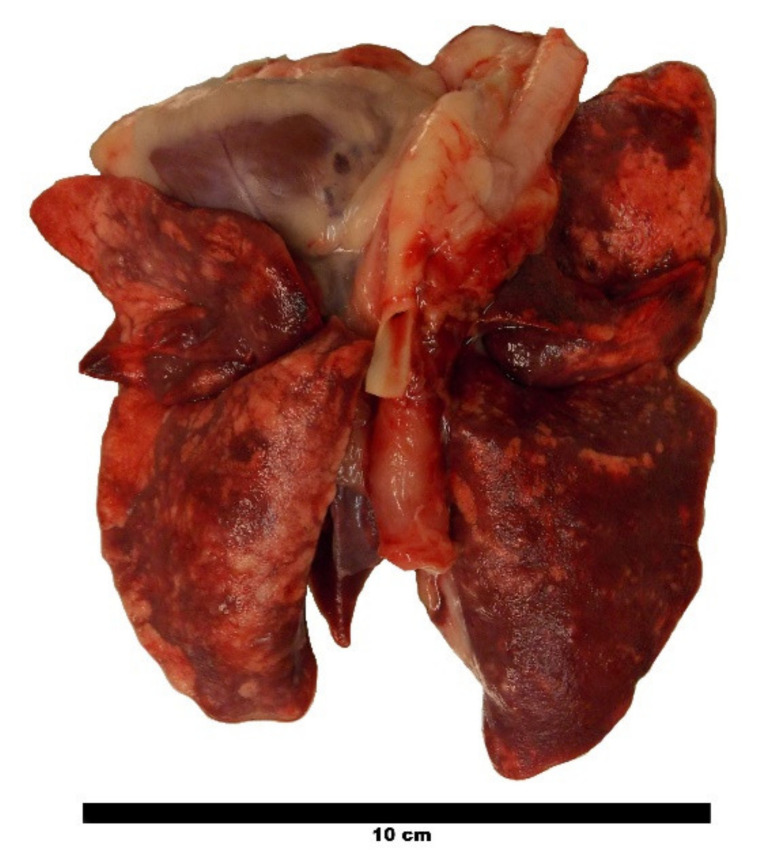
Lungs of cat A3 deceased 168 dpi. The parenchyma of the lungs is interspersed with nodular areas of consolidation, emphysema and firm nodules protruding over the surface of the lungs.

The lungs of cat A3, which deceased 168 dpi due to reasons unrelated to the lungworm infection, showed severe macroscopic visible pathological alterations, such as multifocal areas of consolidation, emphysema and firm nodules protruding over the surface of the lungs (Figure 5). Six weeks before, the same lungs appeared severely altered in CT by a mixed pattern, including consolidated areas and hardly differentiated nodules, reticular to mosaic-like changes, and bronchial wall thickening. The dissection of the lungs revealed a total of 35 adult *A. abstrusus*, namely 13 females and 15 males, as well as seven worm fragments representing heads of the worms.

## 3. Discussion

This study provides new data for the diagnosis of feline aelurostrongylosis, even though the number of cats was low and no statistical evaluations were performed. Importantly, the results represent a new basis for designing anthelmintic efficacy studies relying on alternative criteria than worm counts upon necropsy, thus promoting the 3R principles.

The observed prepatency period of 33–47 days was similar to those observed in other experimental *A. abstrusus* studies ranging between 31 and 41 [13,16], or 40 and 50 [36] days, respectively. However, in the latter study, individual faecal samples were collected only from day 40 pi onwards. In comparison to an anthelmintic efficacy study using the same *A. abstrusus* isolate and the same inoculation dose of 300 L3 [16], the maximum LpG values detected in the present study were considerably lower than those of the untreated control animals in the efficacy study, which ranged between 10.8 and 9180.0 LpG with a mean value of 1514.9 LpG. One reason might be that the cats in the efficacy study were about nine months of age upon infection, whereas here, the age of the cats extends from 13 to 105 months. Furthermore, three cats (B1–B3) received L3 that migrated through a Baermann funnel overnight and, therefore, were inoculated one day after snail digestion. This setup was chosen to assess whether this has a comparable infective potential to L3 used immediately after digestion. If so, this additional migration step would help to remove snail tissue residues in the inoculum, which may be the reason for the frequently reported vomiting of cats after inoculation [13,16,36,37,38]. Here, vomiting after inoculation was not observed in any of the cats. This might be because the inoculation was performed under inhalation anaesthesia, while in other studies, a combination of ketamine and α_2_-agonists is commonly used, and the latter may stimulate emesis. Overall, inoculations with *A. abstrusus* L3 migrated overnight resulted in patent infections in all three cats, but maximum LpG numbers reached only up to one-fifth of those cats inoculated with freshly digested L3. Interestingly, one animal in the latter group did not become patent at all. Hence, further studies including a higher number of animals are required to draw reliable conclusions on the infectivity of *A. abstrusus* L3 isolated with different migrating timing and methods.

The ELISA used for the detection of *A. abstrusus* anti-MSP antibodies showed a sensitivity of 100% in 21 experimentally infected cats 10 wpi [28]. However, in this study, only five of the six animals turned seropositive, and seroconversion required longer than 10 weeks, i.e., 11 and more than 16 weeks in two cats. Interestingly, the cat showing the earliest, but shortest seropositivity from 3 to 4 wpi, remained negative for L1 excretion. This animal may have been infected with male worms only, or larvae excretion was very sporadic and/or in very low numbers, thus remaining unnoticed. Although false-positive ELISA results cannot be ruled out, seropositivity in this cat is supported by moderate to severe alterations in diagnostic imaging, which improved and finally resolved after anthelmintic treatment. Another cat turned seropositive five days before the first L1 detection, while the three other cats were already excreting L1 prior to seroconversion, one of them even for more than 12 weeks, albeit in small numbers. Overall, the course and levels of the measured OD values correlated quite well with those of the LpGs, indicating that high L1 excretion is related to the presence of many male worms or high MSP release due to frequent copulations, respectively. Noteworthy, one of the patent cats remained seronegative throughout the study. Very young animals may remain seronegative despite patent infection, as previously observed [28], suggesting that immunosuppressive conditions may impair antibody production [33]. However, this study animal was 13 months old and there was no evidence for an immunosuppressive state. As larvae excretion of this cat was very low, given the correlation between OD and LpG levels, the most plausible explanation is that the release of MSP was too low to stimulate a detectable antibody production.

After anthelmintic treatment, the OD values declined more or less pronounced as previously reported [28], but only two of the six study cats, both showing marked L1 excretion, were still seropositive at the first treatment date. One of these cats turned seronegative after eight weeks, while the other cat remained seropositive until the end of blood sampling 39 weeks later. Interestingly, a more or less obvious renewed increase in OD values was observed beginning at about 5–15 weeks after the first treatment. This may result from a release of MSP by decaying worms. However, an unspecific immunological reaction in response to a massive release of whole worm antigen during decay cannot be excluded.

Regarding respiratory parameters, there was no significant increase in the respiratory frequency over the course of infection. However, respiratory data were not obtained until day 18, or, in some animals, day 25 pi. Thus, a shortcoming of the study is that an early increase in the respiratory frequency, e.g., when *A. abstrusus* L3 reaches the lung and penetrates into the parenchyma [38], could have been missed. In addition, the respiratory frequencies may be skewed to a certain extent, as the cats became excited or started purring when they became aware of the examiner. Nevertheless, previous studies did not find statistically significant differences as well, though reporting a trend to an increased respiratory frequency in *A. abstrusus* infected cats [13,16]. Distinct clinical signs such as coughing were observed only in three of the cats, and only on single occasions. In pet cats, this feature would have been easily missed by the owner, and if noticed, it is questionable whether they would have been presented to a veterinarian, all the more so as none of the cats included in this study was impaired in general appearance or behaviour. However, such largely inapparent aelurostrongylosis can result in serious disease due to secondary infections with other respiratory pathogens.

Overall, the unremarkable clinical status, as well as the discordant results of the faecal examination and ELISA in this study, was surprising, since all cats showed marked pulmonary changes in radiography and CT with severe findings after infection. The absence of pre-existing aberrances in the lungs was confirmed by CT and radiography immediately before infection. In cat A2, the mild bronchial pattern that was noticed in radiography, but not in CT, was most likely an artefact caused by superimposition of bronchi. The lung alterations visible upon CT examinations after infection were generally consistent with previous findings [19,20], but to the best of our knowledge, this is the first study describing the overtime development of lung change severity in comparison to radiographic severity progression [19] and after anthelmintic treatment. The lung changes in both imaging modalities differed individually to a high degree with an emphasis on specific patterns, but mostly with a mixed appearance and comparable overall severity. Nodular patterns were less visible than expected on the CT, but some small nodules might have been masked by other areas of high attenuation and therefore were lost in the overall mixed appearance of confluent areas of different opacity.

The radiographic lung changes after infection were also consistent with previous descriptions [11,17,20,39], but milder than those visible in the CT in the present study. On the one hand, this could be due to the different scoring systems themselves. In contrast to the CT score system, a maximum score could not be reached in radiography, as long as not all patterns were observed. In this study, there was no overall radiographic severity score based only on the predominant pattern, unlike in other studies [40]. Instead, the score was described as a summary of each pattern score [41], considering the overall variable appearance. On the other hand, mild changes and smaller structures are usually less visible in thoracic radiographs than in CT, as already proven in other studies [20,41,42]. Just as with metastatic disease, small nodular changes are often overlooked in thoracic radiographs but more reliably visible on CT images [43,44]. A subjective component remains when comparing both scores. The latter was adapted from a study examining *Angiostrongylus vasorum* infection in dogs [45], but slightly modified to fit for findings of *A. abstrusus* in cats. This scoring system is less detailed and more subjective than those published before [19,20], yet less time consuming and easier to perform. As previous publications [19,20] did not show significant pulmonary artery changes in their cohorts, no pulmonary artery angiography was included in the present study. There were no notable changes in artery diameters, but the increased opacity of the peribronchiovascular region, which often occurred in the affected lungs, must be taken into consideration. Due to the missing angiographic CT phase, filling defects suggestive of aelurostrongylosis-associated arterial thrombosis cannot be ruled out. In addition, the described peribronchiovascular effacement in some cases impaired the estimation of bronchial wall thickness. Another limitation regarding the diagnostic imaging part of the present study is that there was only a single observer. It remains unclear if inter-observer variability would have led to significantly different scoring results in one or both imaging modalities.

No obvious differences in diagnostic imaging were found between the six cats in this study, and no clear correlation of imaging severity scores and L1 excretion, ELISA OD values or the results of respiratory assessments was notable. In particular, the cats showing no or only low larvae shedding and seroconversion exhibited severe lung changes. A possible explanation for the apparent contradiction between pronounced alterations in diagnostic imaging on the one side, and low L1 excretion and OD values on the other side, might be a strong (not antibody-mediated) immune response. This could have induced marked lung changes but impaired the maturation and reproduction of lungworms and, consequently, the production of anti-MSP antibodies.

When a veterinary surgeon is confronted with the listed imaging findings on CT or thoracic radiographs, it is important to consider a feline lungworm infection as a differential diagnosis. Of course, the diagnosis of aelurostrongylosis cannot rely only on diagnostic imaging findings alone. These are highly suggestive, but there is overlap with other diseases, both non-parasitic (e.g., feline asthma, feline chronic lower airway disease, pulmonary neoplasia or fibrosis, tuberculosis, fungal disease, etc.) and parasitic aetiology, such as *Dirofilaria immitis* [46] and *Toxocara cati* infections [47]. However, in other pulmonary parasitoses, a congestion of pulmonary arteries was reported [46,47] that was neither observed in the presented study nor in a study with naturally infected cats [20]. The same is true for pulmonary hyperinflation associated with peribronchial or alveolar infiltrates often seen in feline asthma. To narrow down the list of differential diagnoses, it is important to inquire about an *A. abstrusus* infection risk (outdoor access with potential ingestion of gastropods and/or small preys) during history taking. However, if faecal examinations remain negative, an infection cannot be ruled out completely, as confirmed in this study; therefore, serology may furnish additional diagnostic support. If other differential diagnoses are unlikely, anthelmintic treatment with proven efficacy against *A. abstrusus* can be applied. As demonstrated in this study, the lung alterations in diagnostic imaging will resolve within 6 to 24 weeks.

The outcome of this study shows that a multiple diagnostic approach is effective to evaluate existing lungworm infections in experimental settings and offers new approaches in the design of anthelmintic efficacy studies. To date, the reduction in adult worm counts inferred from parasitological necropsies is the main efficacy parameter required by the current guidelines [48,49,50], and, therefore, also used in efficacy studies with regard to *A. abstrusus* [16,36,37]. Combining copromicroscopical and serological methods with clinical assessment and diagnostic imaging to obtain a comprehensive picture of L1 excretion, antibody pattern, and the occurrence and resolution of clinical signs and imaging findings allows to confirm (i) an adequate infection of the untreated control animals with sufficient accuracy and (ii) the success of treatment without the need to sacrifice the animals. Following the 3R principles, this concept would be, as already called for [51], a desirable and contemporary alternative to the traditional, and in some aspects, outdated approaches in anthelmintic efficacy studies.

## 4. Materials and Methods

### 4.1. Study Animals

Six purpose-bred male European Shorthair cats, aged between 13 and 105 months (cf. Table S2), free of helminth infections at the start of the study and owned by the Institute for Parasitology, University of Veterinary Medicine Hannover, were included in the study. Animal husbandry and experiments were performed in accordance with the German Animal Welfare act in addition to national and international guidelines for animal welfare. Experiments were permitted by the ethics commission of the Animal Care and Use Committee of the German Lower Saxony State Office for Consumer Protection and Food Safety (*Niedersaechsisches Landesamt für Verbraucherschutz und Lebensmittelsicherheit*) under reference number 33.8-42502-05-17A206.

### 4.2. Infection Material and Experimental Inoculation

The infection of snails (*Cornu aspersum*) with *A. abstrusus* L1 and their husbandry until development of L3 was performed at the University of Teramo, Italy, as previously described [52]. To recover infective L3, the snails were digested according to an established protocol [16] at the Institute for Parasitology, University of Veterinary Medicine Hannover, either on the day of (for cats A1–A3) or the day before (for cats B1–B3) the experimental infection. In the latter case, the L3 were deposited onto a gauze-covered Baermann funnel filled with RPMI 1640, 100 U/mL penicillin, 100 μg/mL streptomycin and 0.25 µg/mL amphotericin B (PAN-Biotech GmbH, Aidenbach, Germany), and larvae migration was allowed overnight at 37 ℃ to eliminate residues of snail tissue. The larvae suspension obtained from both approaches was then placed on a magnetic stirrer and five aliquots of 100 µL were counted to calculate the suspension volume containing 300 L3. The inocula were kept at 20–25 ℃ until experimental application. Inoculation of inhalation-anaesthetised cats (cf. 4.5.1) was performed via a stomach tube immediately after the first CT and X-ray examinations. To avoid vomiting or regurgitation, 0.3 mg/kg metoclopramide (Metomotyl 5 mg/mL, CP-Pharma GmbH, Burgdorf, Germany) was injected intramuscularly approximately 15 min before inoculation. The cats were closely monitored for vomiting for 1 h post inoculation to perform re-inoculation if necessary. No vomiting occurred, neither within one hour after inoculation nor until the next day.

### 4.3. Faecal Larvae Counts

Starting at 32 or 33 dpi, individual faecal samples were collected from each cat three times a week and processed with the Baermann method on the same day. L1 were allowed to migrate overnight at room temperature and the larvae were counted the next morning or, in case of samples collected on Fridays, on the following Monday morning. Until the first detection of L1, the complete faecal sample was used. If a larger number of L1 was detectable, exact amounts of faeces (2–10 g) were weighed out to calculate the number of larvae per gram of faeces (LpG). When the LpG dropped below 0.1, again, the complete faecal sample was used. In the 29th wpi (two weeks after the second anthelmintic treatment, cf. 4.7) sampling was reduced to once a week until the 37th to 39th wpi.

### 4.4. Respiratory Assessment and General Health Monitoring

All cats were observed for general health at least once a day by qualified and trained animal caretakers. Starting between 18 and 25 dpi, and thereafter twice weekly until 36 wpi, all cats underwent a clinical examination of the respiratory system by a veterinary surgeon to assess the following parameters: respiratory frequency, the intensity of respiratory sounds (0 = no sound; 1 = slight sound; 2 = moderate sound; 3 = severe sound), and sound quality (physiological sound, increased respiratory depth, stertor, stridor, rhonchus, wheeze, crackle). In addition, abdominal involvement, panting and coughing or retching were recorded if present.

### 4.5. Anti-MSP Antibody ELISA

For the detection of *A. abstrusus* anti-MSP antibodies, serum samples were collected once before experimental infection by venipuncture of the *Vena cephalica antebrachia* or *Vena saphena medialis*, and afterward, once a week until the end of the faecal sampling at approximately 36 wpi. However, cat A1 was sampled until approximately 63 wpi, since its OD value still exceeded the ELISA cut-off at 36 wpi. Additionally, the four remaining cats (A2, B1–B3; A3 deceased at day 168 pi, cf. 2.1) were sampled once between 51 and 53 wpi as a follow-up. The serum samples were stored at −20 ℃ until shipment to the Institute of Parasitology, Vetsuisse Faculty, University of Zurich, for ELISA analysis following an established protocol using Immobilizer™ Amino-plates (Nunc, Roskilde, Denmark) and HRP-coupled conjugate [32]. The cut-off was set to 0.166 OD, calculated by the mean OD values of the first three samples (collected 1–3 days before and 7 and 14 days after infection) of each cat, plus three standard deviations.

### 4.6. Diagnostic Imaging

#### 4.6.1. Anaesthesia

After placing an intravenous catheter in the right cephalic vein, the cats were premedicated with 0.3 mg/kg midazolame (MIDAzolam, B. Braun Melsungen AG, Melsungen, Germany) and 0.15 mg/kg levomethadone combined with 0.0075 mg/kg fenpipramide (L-Polamivet^®^, Intervet Deutschland GmbH, Unterschleißheim, Germany). Anaesthesia was then induced with propofol (Narcofol^®^, CP-Pharma GmbH, Burgdorf, Germany) to effect intravenously. The cats were intubated and connected to a breathing circuit. Maintenance was performed with isoflurane (Isofluran CP^®^, CP-Pharma GmbH, Burgdorf, Germany) in 100% oxygen. CO_2_ values were kept between 35 and 45 mmHg by mechanical ventilation.

#### 4.6.2. CT Image Acquisition

For computed tomography examinations at different time points during the study (Table S2), the cats were positioned in sternal recumbency. The scans were performed with a 64-slice helical CT scanner (Philips Brilliance 64, Philips Healthcare, Amsterdam, The Netherlands). The image acquisition parameters were as follows: 120 kVp, 150 mAs, rotation time 0.5 s, and collimator pitch 1. The scan field was optimized individually to include the whole lung field. Two pre-contrast scans were performed, one in inspiration with closed pressure valves at 15 cmH_2_O, and one in expiration. The contrast medium (Xenetix^®^ 350 mg/mL, Guerbet GmbH, Sulzbach, Germany) was then administered at a dose of 700 mg/kg over 30 s with a power injector. A bolus tracking technique was used, with a region of interest (ROI) placed in the thoracic aorta. When the measured value in the aorta exceeded a 100 Hounsfield Units (HU) difference, the post-contrast scan was performed after a post threshold delay phase of 20 s. All scans were acquired in apnoea. Reconstruction parameters were as follows: 0.67 mm slice thickness in a 512 × 512 matrix, overlap 0.33 mm, in a soft tissue algorithm (window level 60, window width 400) and a lung algorithm (window level −600, window width 1600). The images were transferred to a dedicated workstation (Philips Brilliance Workspace 3.0) and multiplanar reconstructions were reviewed as described below.

#### 4.6.3. Radiography

Following CT, thoracic radiographs were obtained in left to right latero-lateral and in ventro-dorsal projection.

#### 4.6.4. Image Assessment

All CT and radiography studies were reviewed by a veterinarian with a German Board specialist certification in diagnostic imaging. At the time of image acquisition and image assessment, the veterinarian knew that the cats were infected with *A. abstrusus*, but no data on the course of infection, clinical signs or the date of treatment were disclosed.

The CT lung findings of each cat were recorded and classified using a previously described system for lung assessment [19,45], modified to fit for feline lungworm image assessment. The lungs were divided into three zones, with the first zone being the 1 mm zone at the periphery of each lung lobe; the second zone describing 5% of the lobar width beneath the visceral pleura; the third zone describing the remaining lung parenchyma, including the peribronchovascular regions. The presence of the following lung findings and patterns was recorded for each zone: linear and reticular pattern, nodules or general nodular pattern, areas of high attenuation not described by nodules (e.g., ground-glass opacity, consolidation, marked or severe atelectasis), low attenuation (air trapping, cystic lesions, bullae, bronchiectasis, emphysema), mosaic attenuation pattern (patchwork of areas of different attenuation describing an unstructured interstitial pattern). The appearance of parenchymal bands was also recorded. Bronchial and mediastinal lymph nodes were assessed, and enlargement was noted if present. The bronchial wall thickness was evaluated subjectively and described as normal or thickened. Finally, each lung was scored for the severity of total changes in CT by the use of a previously published [45] and slightly modified scoring system as a guideline, shown in Table 2.

The radiographs were evaluated using a previously described scoring system [14,40,41] with individual scoring of lung patterns: bronchial, alveolar and reticular interstitial patterns, each scored from 0 (absent) to 3 (severe), and nodular interstitial patterns, scored as 0 (absent) or 1 (present). These numbers were then summarized to an individual overall radiographic severity score.

### 4.7. Parasitological Necropsy

One cat (A3) died on day 168 pi due to a reason unrelated to the lungworm infection. Parasitological necropsy of the lungs to count pre-adult *A. abstrusus* was performed as described previously [16].

### 4.8. Anthelmintic Treatment

The remaining cats (A1–A2, B1–B3) were dewormed at day 171 or 173 pi (cf. Table S2), using an emodepside/praziquantel combination (Profender ^®^ spot-on for cats multi-dose bottle, then Bayer Animal Health GmbH, Leverkusen, Germany, now Vetoquinol GmbH, Ismaning, Germany) at a dosage of 3 mg/kg emodepside and 12 mg/kg praziquantel. According to the recommendations of the manufacturer, the treatment was repeated after 14 days.

## 5. Conclusions

The diagnosis of an aelurostrongylosis in cats remains challenging, especially when faecal larvae excretion is absent. Diagnostic imaging can be a helpful tool, where CT allows a better and more detailed examination of lung alterations compared to radiographs. However, the changes are variable in appearance and not pathognomonic, requiring further diagnostics. Anti-MSP antibody detection by ELISA could support diagnostics as well, but, as with the Baermann method, has limitations when worm reproduction is low. A multi-diagnostic approach is therefore warranted, combining L1 counts, serology, clinical parameters and diagnostic imaging. This approach is not only important to be considered in a clinical setting, but also provides the basis for an innovative 3R-compliant concept for designing anthelmintic efficacy studies without the need for *post mortem* worm counts.

## Figures and Tables

**Table 1:** Computed tomography (CT) and thoracic radiographs (XRAY) lung severity score results for six cats experimentally infected with *A. abstrusus*.

Cat	Imaging Modality	Week Post Infection							
		0	12	18/19th	25th *	30th/31th	36th/37th	42	48th
A1	CT	0	3	2	2	0			
	XRAY	0	7	6	5	1			
A2	CT	0	2	3	3	1	0		
	XRAY	1	4	4	4	1	0		
A3	CT	0	3	3					
	XRAY	0	5	4					
B1	CT	0	3	2	3	1	0		
	XRAY	0	5	3	5	2	0		
B2	CT	0	2	3	2	1	0		
	XRAY	0	5	5	2	0	0		
B3	CT	0	3	2	2	1	1	1	0
	XRAY	0	5	3	3	2	1	0	0

In CT, the lungs were scored as 0 = normal, 1 = mild, 2 = moderate, or 3 = severely altered. In XRAY, the score scale ranges from 0 = normal to 10 = most severe. The asterisk indicates the first anthelmintic treatment. Note that cat A3 deceased on day 168 pi.

**Table 2:** Computed Tomography (CT) Severity Scoring System. This table displays the score used to describe the overall severity of lung changes seen in the CT examinations of the present study.

CT Lung Severity Score	Imaging Features
0 = normal	No changes
1 = mild	Some or all zones affected, some areas of ground-glass opacity, only occasional nodules or consolidated areas, mild reticular and/or mosaic pattern, no or mild bronchial wall thickening, no lymph node enlargement
2 = moderate	All zones affected, some or multiple areas of ground-glass opacity, multiple or all lobes affected, occasional consolidated areas, no or some nodules, moderate reticular and/or mosaic pattern, bronchial wall thickening, partial loss of visual separation between bronchial walls and peribronchial vessels, enlarged lymph nodes
3 = severe	All zones affected, multiple nodules or general nodular pattern, multiple consolidated areas, severe reticular and/or mosaic pattern bronchial wall thickening, marked ground-glass opacity, loss of visual separation between bronchial walls and peribronchial vessels, enlarged lymph nodes

## Data Availability

Data supporting reported results is contained within the article and the Supplementary Materials.

## Supplementary Materials

The following are available online at https://www.mdpi.com/article/10.3390/pathogens10050602/s1, Table S1: Respiratory Parameters, Table S2: Study data.
