# Supplementary material for: Do All Roads Lead to Rome? The Potential of Different Approaches to Diagnose Aelurostrongylus abstrusus Infection in Cats
Source: Pathogens. 2021 May 14;10(5):602. doi: 10.3390/pathogens10050602 (PMC8157210; doi:10.3390/pathogens10050602)
Supplement: Supplementary file 1 [file pathogens-10-00602-s001.zip › pathogens-1221108-supplementary/Table_S1_Resp.pdf]

Supplementary Table 1. Results of the respiratory assessment during the course of experimental *Aelurostrongylus abstrusus* infection and after anthelminthic treatment with emodepside/praziquantel at days 171/173 (first treatment) and 185/187 pi (second treatment). The intensity of respiratory sounds during auscultation was scored between 0 (no sound) and 3 (severe sound). Abnormal quality of respiratory sounds as well as coughing or retching were noted if observed. The days after all cats received the first treatment are marked in grey. Note that cat A3 deceased on day 168 pi. Abbreviations: dpi = days post infection; - = omitted; IRD = increased respiratory depth; CR = crackle; W = wheeze.

**dpi /**	**Respiratory rate/min**						**Intensity**						**Quality**						**Coughing/retching**					
**Cat**	**A1**	**A2**	**A3**	**B1**	**B2**	**B3**	**A1**	**A2**	**A3**	**B1**	**B2**	**B3**	**A1**	**A2**	**A3**	**B1**	**B2**	**B3**	**A1**	**A2**	**A3**	**B1**	**B2**	**B3**
**18**	-	40	52	-	28	32	-	0	1	-	0	-	-			-			-			-		
**21**	-	28	30	-	36	36	-	0	1	-	1	-	-	CR		-		IRD	-			-	Yes	
**25**	48	36	28	48	52	64	1-2	1-2	1	1	1	1		IRD			IRD	IRD						
**28**	32	28	32	40	66	54	1-2	1	1	1	1	1	IRD			IRD	IRD	IRD						
**32**	40	40	48	28	66	54	1	1	1	1	1	1	IRD				IRD	IRD						
**35**	30	54	84	28	42	60	1	0	1	1	1	1			IRD		IRD	W						
**39**	44	54	48	40	54	48	1	1	1	2	1	1	IRD		IRD	IRD	IRD	IRD						
**42**	54	44	60	42	54	52	1	0	1	1	1	2				IRD	IRD	IRD						
**46**	54	54	48	48	48	48	1	0	1	1	2	1	IRD		IRD	IRD	IRD	IRD						
**49**	42	44	66	36	52	56	1	0	0	1	1	1	IRD			IRD	IRD	IRD						
**53**	44	56	54	52	52	42	1	0	1	1	1	2	IRD		IRD	IRD	IRD	IRD						
**56**	52	66	54	48	60	42	1	1	1	1	1	1		IRD	IRD	IRD	IRD	IRD						
**60**	44	48	44	36	48	52	1	0	1	1	1	1	IRD		IRD	IRD	IRD							
**63**	48	36	48	52	40	48	1	0	1	1	1	1	IRD		IRD	IRD	IRD	IRD						
**67**	44	32	44	48	52	44	1	0	1	1	1	1			IRD		IRD	IRD						
**70**	54	36	64	36	48	48	1	0	1	1	2	2	IRD		IRD	IRD	IRD	IRD						
**74**	40	36	48	60	72	66	1	0	1	1	2	2			IRD		IRD	IRD						
**77**	44	52	60	52	44	52	1-2	0	1	1	1	2	CR			IRD		IRD	Yes					
**81**	40	60	54	44	48	48	1	0	2	1	1	1			IRD	IRD		IRD						
**84**	48	52	56	44	32	40	1	0	2	1	1	1	IRD		IRD			IRD						
**88**	66	32	44	84	48	40	2	0	1	2	1	1	IRD		IRD	IRD		IRD						
**91**	44	36	54	52	60	52	1	0	1	2	1	1	IRD					IRD		Yes				
**95**	44	44	52	32	52	32	1	1	1	1	1	1			IRD	IRD		IRD						

**98**	56	54	48	48	48	44	1	0	1	1	1	1	IRD			IRD	IRD	IRD						
**102**	36	44	48	52	40	44	1	1	1	1	1	1			IRD	IRD		IRD						
**104**	-	-	-	-	60	60	-	-	-	-	1	1	-	-	-	-	IRD	IRD	-	-	-	-		
**105**	54	54	54	60	-	-	0	1	1	1	-	-			IRD	IRD	-	-					-	-
**109**	48	36	52	52	45	44	1	0	1	1	1	1	IRD		IRD	IRD		IRD						
**111**	-	60	72	-	60	68	-	0	1	-	1	2	-		IRD	-		IRD	-			-		
**112**	64	-	-	40	-	-	1	-	-	1	-	-	IRD	-	-	IRD		-		-	-		-	-
**116**	48	40	32	40	40	44	1	0	1	1	1	2	IRD		IRD	IRD		IRD		Yes				
**118**	52	72	66	60	54	54	2	1	1	2	2	2		IRD	IRD			IRD						
**123**	44	36	48	52	48	48	1	1	1	1	1	1-2			IRD	IRD		IRD						
**125**	54	48	54	66	54	44	1	0	2	1	1	1												
**130**	32	32	44	64	52	48	1	0	1	1	1	1	IRD		IRD		IRD	IRD						
**132**	60	46	54	60	40	48	1	1	1	1	1	1					IRD	IRD						
**137**	40	52	48	60	48	52	1	1	1	1	1	1			IRD	IRD	IRD	IRD						
**139**	46	44	48	44	-	-	1	0	1	1	-	-					-	-					-	-
**140**	-	-	-	-	58	48	-	-	-	-	1	0	-	-	-	-	IRD		-	-	-	-		
**144**	32	44	44	44	48	56	0	1	1	1	1	1-2			IRD	IRD		IRD						
**146**	48	-	-	44	-	-	1	-	-	1	-	-	IRD	-	-	IRD	-	-		-	-		-	-
**147**	-	52	48	-	48	44	-	1	0	-	1	1	-	IRD		-	IRD	IRD	-			-		
**151**	48	40	48	52	28	48	1	0	1	1	1	1			IRD	IRD		IRD						
**154**	44	44	52	40	48	52	1	1	1	1	1	1	IRD			IRD								
**158**	44	32	56	32	40	60	1	1	1	1	1	1	IRD	IRD				IRD						
**160**	-	-	-	-	40	60	-	-	-	-	1	0	-	-	-	-	IRD		-	-	-	-		
**161**	56	48	60	48	-	-	1	1	1	1-2	-	-	IRD	IRD		IRD	-	-					-	-
**165**	42	36	48	48	54	66	1	1	1	1	0	0			IRD									
**167**	-	44	48	-	-	-	-	1	0	-	-	-	-	IRD		-	-	-	-			-	-	-
**168**	52	-		48	56	48	1	-		1	0	0		-						-				
**172**	44	42		56	48	42	1	1		1	1	1-2						IRD						
**174**	40	-		64	-	-	1	-		1	-	-	IRD	-		IRD	-	-		-			-	-
**175**	-	40		-	42	32	-	1		-	0	1	-	IRD		-		IRD	-			-		

**179**	66	48		54	66	62	1	1		1	1	1		IRD										
**182**	44	48		40	46	55	1	0		1	0	1	IRD			IRD								
**186**	42	42		60	54	60	0	0		1	1	2				IRD		IRD						
**189**	42	52		32	60	54	0	1		0	1	1												
**193**	46	42		54	66	42	1	1		1	0	0												
**196**	62	48		42	52	48	0	1		0	1	1												
**200**	54	42		60	52	48	1	0		1	1	1												
**202**	-	-		-	62	40	-	-		-	0	0	-	-		-			-	-		-		
**203**	54	44		54	-	-	1	0		1	-	-	IRD				-	-					-	-
**207**	48	40		48	52	44	1	0		1	0	0												
**209**	-	48		-	-	-	-	0		-	-	-	-			-	-	-	-			-	-	-
**210**	44	-		48	40	48	0	-		1	1	1		-						-				
**214**	40	40		36	48	44	0	0		1	1	1												
**216**	44	-		36	-	-	0	-		0	-	-		-			-	-		-			-	-
**217**	-	32		-	54	54	-	0		-	0	0	-			-			-			-		
**220**	-	-		-	44	40	-	-		-	1	1	-	-		-			-	-		-		
**221**	48	44		56	-	-	0	0		1	-	-					-	-					-	-
**224**	32	36		56	62	55	0	0		0	0	0												
**227**	-	36		-	66	60	-	0		-	0	0	-			-			-			-		
**228**	44	-		56	-	-	0	-		1	-	-		-			-	-		-			-	-
**231**	66	54		54	42	48	0	0		0	0	0												
**234**	40	46		56	-	-	0	0		1	-	-					-	-					-	-
**235**	-	-		-	48	44	-	-		-	0	0	-	-		-			-	-		-		
**237**	-	42		-	-	-	-	0		-	-	-	-			-	-	-	-			-	-	-
**238**	46	-		42	54	42	0	-		0	1	1		-						-				
**241**	48	-		50	-	-	0	-		0	-	-		-			-	-		-			-	-
**242**	-	32		-	48	40	-	0		-	1	1	-			-	IRD	IRD	-			-		
**245**	60	42		48	48	56	0	1		0	0	0												
**249**	36	32		40	56	44	0	0		1	1	1						-						
**252**	60	36		48	48	40	1	0		1	1	1						-						
