# Supplementary material for: Do All Roads Lead to Rome? The Potential of Different Approaches to Diagnose Aelurostrongylus abstrusus Infection in Cats
Source: Pathogens. 2021 May 14;10(5):602. doi: 10.3390/pathogens10050602 (PMC8157210; doi:10.3390/pathogens10050602)
Supplement: Supplementary file 1 [file pathogens-10-00602-s001.zip › pathogens-1221108-supplementary/Table_S2_Study data.pdf]

Supplementary Table 2. Detailed study data on the age of the cats at the day of infection, type of inoculum (fresh *Aelurostrongylus abstrusus* L3 from the day of snail digestion or L3 allowed to migrate overnight through a Baermann funnel after snail digestion) as well as data of CT/XRAY examinations and anthelmintic treatment with emodepside/praziquantel. Note that cat A3 deceased on day 168 pi. Abbreviations: L3 = third-stage larvae; CT = computed tomography; XRAY = radiography; dpi = days post infection.

**Cat**	**A1**	**A2**	**A3**	**B1**	**B2**	**B3**
**Age at infection (month)**	37	38	105	37	13	13
**Inoculum**	300 L3 (fresh)	300 L3 (fresh)	300 L3 (fresh)	300 L3 (migrated)	300 L3 (migrated)	300 L3 (migrated)
**1st CT/X-RAY (dpi)**	0	0	0	0	0	0
**2nd CT/XRAY (dpi)**	84	84	84	84	84	84
**3rd CT/XRAY (dpi)**	126	129	129	126	126	126
**4th CT/XRAY (dpi)**	171	173		171	171	171
**1st Anthelmintic treatment (dpi)**	171	173		171	171	171
**2nd Anthelmintic treatment (dpi)**	185	187		185	185	185
**5th CT/XRAY (dpi)**	213	210		213	209	209
**6th CT/XRAY (dpi)**		249		256	252	252
**7th CT/XRAY (dpi)**					294	
**8th CT/XRAY (dpi)**					337	
